# The complete nucleotide sequence and genome organisation of a novel member of the family *Betaflexiviridae* from *Actinidia chinensis*

**DOI:** 10.1007/s00705-017-3701-x

**Published:** 2018-01-29

**Authors:** Stella Veerakone, Lia W. Liefting, Joe Tang, Lisa I. Ward

**Affiliations:** Plant Health and Environment Laboratory, Ministry for Primary Industries, P.O. Box 2095, Auckland, 1140 New Zealand

## Abstract

We report the complete genome sequence of a novel virus, tentatively named “actinidia seed-borne latent virus” (ASbLV), isolated from *Actinidia chinensis* in Auckland, New Zealand. The complete genome of ASbLV is 8,192 nucleotides long, excluding the 3ʹ poly(A) tail, contains four open reading frames, and is most closely related to Caucasus prunus virus (56% nucleotide sequence identity), a member of the genus *Prunevirus*. Based on the demarcation criteria of the family *Betaflexiviridae*, ASbLV is a new member of the genus *Prunevirus*.

**Electronic supplementary material:**

The online version of this article (10.1007/s00705-017-3701-x) contains supplementary material, which is available to authorized users.

The genus *Actinidia* (commonly known as kiwifruit), whose members occur naturally in China, is composed of over 50 species. Of these, *A. deliciosa* and *A. chinensis* are the most widely cultivated for fruit production. Kiwifruit was first introduced into New Zealand from China in the early 1900, and New Zealand is now one of the biggest kiwifruit producers in the world.

In 2013, *A*. *chinensis* vines grown from seeds in a home garden in Auckland, New Zealand, showed virus-like symptoms of vein chlorosis and mottling. Symptomatic leaf samples from five vines were collected, and total RNA was extracted from three bulked leaves per vine, using an RNeasy Plant Mini Kit (QIAGEN, Limburg, The Netherlands) according to the manufacturer’s instructions. The RNA from each vine was tested by reverse transcription polymerase chain reaction (RT-PCR) for a range of viruses, including those previously reported to infect kiwifruit. RT-PCR was performed with various generic and specific primers, using the Invitrogen SuperScript III One-Step RT-PCR System with Platinum *Taq* DNA Polymerase (Life Technologies, Carlsbad, USA). Four of the five vine samples were positive for actinidia virus A using species-specific primers [1]. A 360-bp product was obtained from the fifth vine sample using a generic trichovirus and vitivirus PCR [2]. However, sequence analysis indicated that the replicase protein gene of this virus was phylogenetically closest to that of citrus leaf blotch virus (CLBV, genus *Citrivirus*) but it was distinct from those of all trichoviruses and vitiviruses. A primer pair that amplifies a 425-bp region of the CLBV coat protein gene [3] did not yield an amplicon of the expected size, suggesting the presence of a novel virus. This report summarises the genome organisation of this novel virus.

To determine the complete genome sequence of the novel virus, symptomatic *A*. *chinensis* leaf tissue was homogenised in RLT buffer (QIAGEN) modified according to MacKenzie et al. [4]. Total nucleic acid was extracted from the homogenate using an InviMag Plant DNA Mini Kit (Invitek GmbH, Berlin, Germany) on a Kingfisher mL workstation (Thermo Scientific, Waltham, MA, USA). DNA was removed from the sample by treatment with DNase 1 using an Ambion DNA-*free* Kit (Life Technologies), followed by host ribosomal RNA (rRNA) removal using a Ribo-Zero rRNA Removal Kit for plant leaf (Epicentre, Madison, WI, USA) according to the manufacturer’s instructions. The rRNA-depleted RNA was fragmented and used to synthesise double-stranded cDNA as described in the cDNA Rapid Library Preparation Method Manual, GS Junior Titanium Series, May 2010 (Rev, June 2010; Roche Life Sciences). The sample was sequenced on a 454 GS Junior System (Roche 454 Life Sciences) following the manufacturer’s protocols. A total of 189,817 reads averaging 433 bp in length were obtained from the GS Junior sequencing run.

The GS Junior reads were mapped to reference sequences of actinidia virus B, apricot vein clearing associated virus, Caucasus prunus virus, cherry rusty mottle associated virus, CLBV and prunus virus T. The best reference mapping was to Caucasus prunus virus (CPrV) where the GS Junior reads mapped to the CPrV genome at positions approximately 21 to 1,685 bp and 3,598 to 7,870 bp. Repeated rounds of mapping the GS Junior reads to the consensus sequences extended the contig ends until the complete genome of the novel virus was obtained in a single contig, apart from a small region at the 5ʹ end. Homopolymer regions were resolved by Sanger sequencing of amplicons generated using specific primer sets designed from the contig sequence. Overall, 5,272 GS Junior and Sanger reads were assembled with a mean coverage level of 278 (maximum 447; minimum 47). The remaining 5ʹ end of the genome was determined using a GeneRacer Kit (Life Technologies) as per the manufacturer’s instructions. All sequence analysis was performed using Geneious 10.0.6 (Biomatters Ltd, Auckland, New Zealand).

The complete genome sequence of the novel *Actinidia* virus consists of 8,192 nucleotides (nt) (GenBank accession number MF440375), excluding the 3ʹ poly(A) tail (Fig. 1A). Four open reading frames (ORFs) were predicted using the ORF Finder tool at the National Center for Biotechnology Information (NCBI): ORF1 (nt 66-6074) encodes a putative replicase (Pol) of 2,002 amino acids (aa), ORF2 (nt 6067-7407) encodes a putative movement protein (MP) of 446 aa, ORF3 (nt 6983-7651), which overlaps ORF2 by 424 nt, encodes a putative coat protein (CP) of 222 aa, and ORF4 (nt 7653-8126) encodes a putative nucleic acid binding protein (NB) of 157 aa. The genome organisation is similar to that of members of the genus *Prunevirus*, a new genus of the family *Betaflexiviridae* [7], and to some members of the genus *Trichovirus* (cherry mottle leaf virus and peach mosaic virus).

**Fig. 1:**
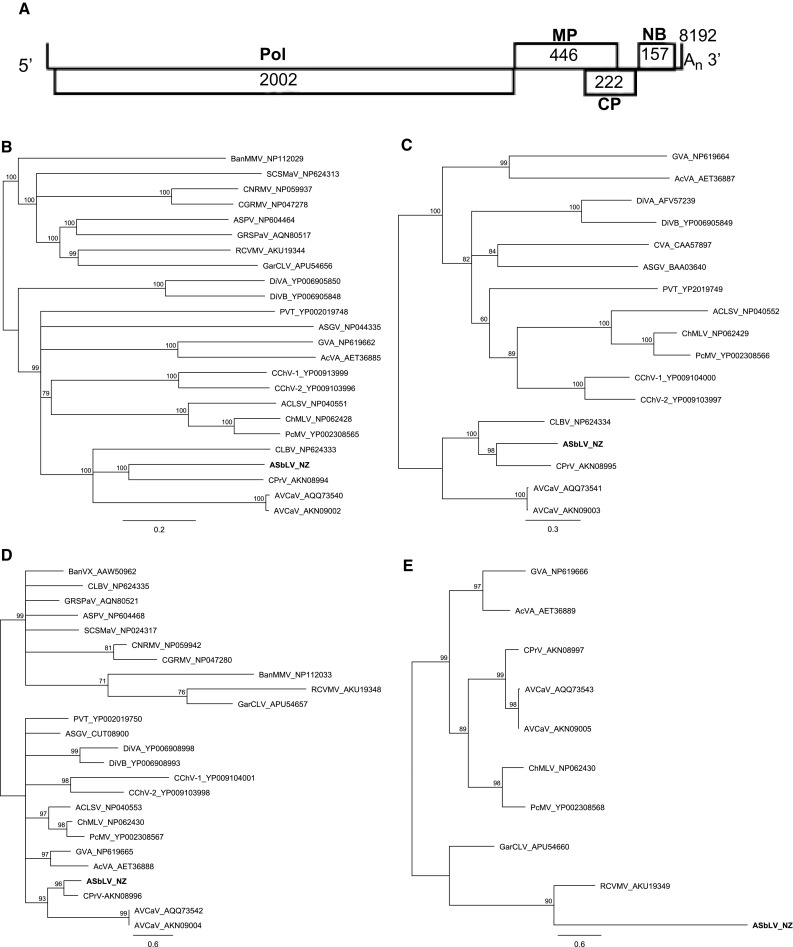
Characterisation of a novel *Actinidia* virus, a new member of the family *Betaflexiviridae*. (A) Schematic representation of the genomic organisation. The locations of the replicase (Pol), movement protein (MP), coat protein (CP) and nucleic acid binding protein (NB) and their amino acid sizes are indicated. (B, C, D and E) Phylogenetic analysis of representative members of the family *Betaflexiviridae* based on the deduced amino acid sequences of the replicase (B), movement protein (C), coat protein (D) and nucleic acid binding protein (E). The abbreviations of the virus names are given in Online Resource 1. Branches with more than 60% bootstrap support from 1000 replicates are shown

The genus *Prunevirus*, subfamily *Trivirinae* includes two species, *Apricot vein clearing associated virus* and *Caucasus prunus virus*. Members of both species have been found to infect trees of the genus *Prunus*. The genomes of these viruses contain four ORFs. ORF1 encodes a large polypeptide that functions as a replicase, ORF2 encodes the 30K-type MP, ORF3 encodes the CP, and ORF4 encodes the NB. Members of this genus show affinities with members of the genus *Citrivirus* in the replicase and MP genes. However, the 3ʹ portion of their genome differs from that of citriviruses by possessing an NB ORF and showing different phylogenetic affinities in the CP gene; citriviruses are closer to the triple gene block group, whereas prunevirus are closer to the 30K-type group [5].

The complete genome sequence and the deduced aa sequences encoded by ORF1, ORF2, ORF3 and ORF4 of the novel *Actinidia* virus were compared separately with sequences of other viruses belonging to the family *Betaflexiviridae.* Phylogenetic analysis of aa sequences was performed using Geneious 10.0.6. The neighbour-joining (NJ) method was used to construct the phylogenetic trees with the Jukes-Cantor model, and the statistical significance of the branches was evaluated with 1000 bootstrap replications. Phylogenetic analysis of the deduced aa sequences of the different genes showed that the novel *Actinidia* virus clusters with the members of the genus *Prunevirus* (Fig. 1B-D) with the exception of the NB gene (Fig. 1E), which clusters with some members of the genus *Carlavirus* (red clover vein mosaic virus and garlic common latent virus). Comparison of the novel *Actinidia* virus Pol, MP and CP aa sequences with those of the other members of the family *Betaflexiviridae* showed sequence identities ranging from 20 to 47%, 8 to 52% and 6 to 64%, respectively; seven viruses with the highest identity are shown in Table 1. All three proteins showed highest aa identity (47.1%, 52.3% and 63.5%, respectively) to the corresponding proteins of the Caucasus prunus virus (CPrV) (Table 1). Similarly, the complete genome sequence of the novel *Actinidia* virus showed the closest similarity to that of CPrV, with 56% nt sequence identity. This is well below the threshold value (less than 80% identity at the aa level in the CP or replicase genes) used as the molecular criterion for species demarcation, and above the value (less than 45% identity at the nt level) used for genus demarcation throughout the family *Betaflexiviridae*.

**Table 1:** Percentage identity of the deduced amino acid (aa) sequences of the Pol, MP, CP, and NB and the nucleotide (nt) sequence of the complete genome of the novel *Actinidia* virus to some other members of the family *Betaflexiviridae*

Virus	Pol (aa)	MP (aa)	CP (aa)	NB (aa)	Genome (nt)
CPrV	47.1	52.3	63.5	9.5	56.1
AVCaV	37.8	34.3	32.5	10.1	48.6
CLBV	37.7	38.1	7.2	na	45.9
RCVMV	23.5	na	12.3	27.9	36.7
GarCLV	21.6	na	10.9	22	34.5
ChMLV	24.7	12.5	25.5	12.7	39.4
PcMV	24.3	10.8	24.2	12	38.9

Virus abbreviations are given in Supplementary Table 1

Based on the Pol, MP and CP genes, the new virus is most closely related to, but distinct from, CPrV. The genetic difference in the NB gene suggests a possible recombination event. Genome recombination has been frequently identified in members of the family *Betaflexiviridae* [6, 7].

Based on the genome organisation and the degree of sequence similarity to known members of the genus *Prunevirus*, the newly discovered virus should be considered a member of the genus *Prunevirus*, which we tentatively name “actinidia seed-borne latent virus” (ASbLV). The original vine and three more vines (tested later) from the same property were co-infected with actinidia virus A (genus *Vitivirus*) and/or actinidia virus 1 (family *Closteroviridae*). Although the plants in which the virus was detected were symptomatic, ASbLV was also detected in asymptomatic vines as a single infection. Assessment of the biological properties of this virus is challenging due to the difficulty in transmitting the virus to herbaceous indicators. The impact of ASbLV is currently being investigated by Plant and Food Research (P&F) scientists. Other viruses belonging to the family *Betaflexiviridae* reported in *Actinidia* sp. include actinidia citrivirus, actinidia virus A, actinidia virus B and apple stem grooving virus (ASGV). All of these viruses except ASGV have been reported only in kiwifruit. To date, no efficient vectors have been reported for these viruses, which are thought to be transmitted only by grafting [1].

## Electronic supplementary material

Below is the link to the electronic supplementary material.

Supplementary material 1 (PDF 29 kb)

Supplementary material 2 (FASTA 8 kb)
