# Supplementary material for: The complete nucleotide sequence and genome organisation of a novel member of the family Betaflexiviridae from Actinidia chinensis
Source: Arch Virol. 2018 Jan 29;163(5):1367–70. doi: 10.1007/s00705-017-3701-x (PMC5886997; doi:10.1007/s00705-017-3701-x)
Supplement: Supplementary file 1 — Supplementary material 1 (PDF 29 kb) [file 705_2017_3701_MOESM1_ESM.pdf]

The complete nucleotide sequence and genome organisation of a novel *Betaflexiviridae* from *Actinidia chinensis*

Archive of Virology

Stella Veerakone, Lia W. Liefting, Joe. Tang, Lisa I. Ward

Plant Health and Environment Laboratory, Ministry for Primary Industries, P.O. Box 2095, Auckland 1140, New Zealand

Email: stella.veerakone@mpi.govt.nz

Table 1 List of virus species used for the phylogenetic analysis

Virus	Acronym	Genus	Subfamily
Red clover vein mosaic virus	RCVMV	Carlavirus	Quinvirinae
Garlic common latent virus	GarCLV	Carlavirus	Quinvirinae
Apple stem pitting virus	ASPV	Foveavirus	Quinvirinae
Grapevine rupestris stem pitting-associated virus	GRSPaV	Foveavirus	Quinvirinae
Cherry necrotic rusty mottle virus	CNRMV	Robigovirus	Quinvirinae
Cherry green ring mottle virus	CGRMV	Robigovirus	Quinvirinae
Banana mild mosaic virus	BanMMV	Unassigned	Quinvirinae
Banana virus X	BanVX	Unassigned	Quinvirinae
Sugarcane striate mosaic-associated virus	SCSMaV	Unassigned	Quinvirinae
Apple stem grooving virus	ASGV	Capillovirus	Trivirinae
Cherry virus A	CVA	Capillovirus	Trivirinae
Carrot Ch virus 1	CChV-1	Chordovirus	Trivirinae
Carrot Ch virus 2	CChV-2	Chordovirus	Trivirinae
Citrus leaf blotch virus	CLBV	Citrivirus	Trivirinae
Diuris virus A	DiVA	Divavirus	Trivirinae
Diuris virus B	DiVB	Divavirus	Trivirinae
Apricot vein clearing associated virus	AVCaV	Prunevirus	Trivirinae
Caucasus prunus virus	CPrV	Prunevirus	Trivirinae
Potato virus T	PVT	Tepovirus	Trivirinae
Apple chlorotic leaf spot virus	ACLSV	Trichovirus	Trivirinae
Cherry mottle leaf virus	ChMLV	Trichovirus	Trivirinae
Peach mosaic virus	PcMV	Trichovirus	Trivirinae
Actinidia virus A	AcVA	Vitivirus	Trivirinae
Grapevine virus A	GVA	Vitivirus	Trivirinae
